# Safety of In-hospital Parenteral Antiosteoporosis Therapy Following a Hip Fracture: A Retrospective Cohort

**DOI:** 10.1210/jendso/bvae172

**Published:** 2024-10-07

**Authors:** Alaa Abu-Jwead, David L Fisher, Adi Goldabart, Uri Yoel, Yan Press, Anat Tsur, Merav Fraenkel, Lior Baraf

**Affiliations:** Goldman Medical School at the Faculty of Health Sciences, Ben-Gurion University of the Negev, Beer Sheva 8410501, Israel; Endocrinology, Soroka University Medical Center, Beer Sheva 84101, Israel; Clinical Research Center, Soroka University Medical Center, Beer Sheva 84101, Israel; Endocrinology, Soroka University Medical Center, Beer Sheva 84101, Israel; Faculty of Health Sciences, Ben-Gurion University of the Negev, Beer Sheva 8410501, Israel; Geriatric Department, Soroka University Medical Center, Beer Sheva 84101, Israel; Faculty of Health Sciences, Ben-Gurion University of the Negev, Beer Sheva 8410501, Israel; Department of Endocrinology and Metabolism, Clalit Health Services, Jerusalem 9310604, Israel; The Faculty of Medicine, Hebrew University of Jerusalem, Jerusalem 9190500, Israel; Endocrinology, Soroka University Medical Center, Beer Sheva 84101, Israel; Faculty of Health Sciences, Ben-Gurion University of the Negev, Beer Sheva 8410501, Israel; Endocrinology, Soroka University Medical Center, Beer Sheva 84101, Israel; Faculty of Health Sciences, Ben-Gurion University of the Negev, Beer Sheva 8410501, Israel

**Keywords:** osteoporosis, hip fracture, zoledronic acid, denosumab, adverse effects

## Abstract

**Purpose:**

To assess the safety of zoledronic acid (ZOL) and denosumab (Dmab) administered following hip fracture in a hospital setting.

**Methods:**

Patients older than 65 years were treated by a fracture liaison service following hip fracture. Generally, patients who had a glomerular filtration rate (eGFR) > 35 mL/min were treated with ZOL, whereas patients who had previously received bisphosphonates or had a eGFR between 20 and 35 mL/min were treated with Dmab. Adverse events included hypocalcemia (calcium corrected for albumin less than 8.5 mg/day), renal functional impairment (0.5 mg/dL or more increase in serum creatinine) within 30 days of treatment, or a fever (>38 °C) within 48 hours of drug administration.

**Results:**

Two hundred twenty-eight and 134 patients were treated with ZOL and Dmab, respectively. Mean body temperature was elevated following ZOL administration (0.18 °C *P* < .001) but remained below 38 °C. Hypocalcemia occurred in 18% and 29% of the ZOL and Dmab groups, respectively (*P* = .009). Renal functional impairment was observed in 9 and 6 patients (4% and 5%) in the ZOL and Dmab groups, respectively (*P* = .8). Pretreatment calcium above 9.3 mg/dL was associated with a lower risk of posttreatment hypocalcemia (odds ratio 0.30, 95% confidence interval 0.13-0.68, *P* = .004). While the absolute risk of hypocalcemia was higher in the Dmab group, multivariate analysis did not find that the choice of drug was predictive of hypocalcemia.

**Conclusion:**

In-hospital parenteral osteoporosis treatment was rarely associated with fever or renal function impairment but was associated with hypocalcemia. Posttreatment hypocalcemia risk did not vary significantly between patients receiving ZOL or Dmab.

Osteoporotic hip fractures usually affect elderly populations and have an increasing global prevalence [1]. The risk of mortality increases 3- to 4-fold during the first year following a hip fracture, and the risk of suffering from another osteoporotic fracture is also increased, although this risk is attenuated by postfracture osteoporosis treatment [2]. Zoledronic acid (ZOL) or denosumab (Dmab) are first-line treatments following hip fracture [3]. The HORIZON study demonstrated that treatment with ZOL following a hip fracture reduces the risk of subsequent nonvertebral, clinical fractures and clinical vertebral fractures by 25%, 33%, and 77%, respectively, and reduces mortality in the first year following fracture [4]. The FREEDOM extension trial showed that treatment with Dmab for 10 years increased bone mineral density and was associated with a reduced incidence of vertebral fracture by 68% and hip fracture by 40% [5].

Three major adverse events (AE) have been described following treatment with ZOL or Dmab: flu-like symptoms, hypocalcemia, and acute kidney injury [4, 6-9]. Flu-like symptoms such as transient fever and myalgia occur as part of an acute-phase response (APR). These symptoms are well recognized following ZOL administration [6] but have not been observed following Dmab treatment. Symptoms are more common after the first dose of ZOL compared with subsequent administrations [6]. Fever usually starts within 24 hours of ZOL infusion and subsides after 2 to 3 days [7]. Prior treatment with antipyretic therapy reduces the risk of fever [10]. Hypocalcemia may occur following administration of ZOL or Dmab [4, 8]. It is thought to result from osteoclast inhibition, which reduces calcium mobilization from bone matrix [11]. Hypocalcemia occurs more profoundly following Dmab injection compared with ZOL infusion [12]. Dmab-induced hypocalcemia may also be mediated via an increase in cortical bone formation via a modeling-based mechanism [13], and activation of osteoblasts has also been postulated as another possible mechanism for hypocalcemia after ZOL treatment [14]. Renal function impairment is a rare but well-documented AE following ZOL administration [15]. Most cases have been reported in patients with multiple myeloma or advanced solid cancer who received ZOL more frequently as compared to patients with osteoporosis [16]. In patients with osteoporosis with an estimated glomerular filtration rate (eGFR) greater than 30 mL/min, who receive yearly ZOL infusions, a transient and mild impairment of renal function has been rarely reported that does not lead to chronic renal disease [9]. Prehydration and infusing the drug at a slow rate (over longer than 15 minutes) may be protective [17].

Fracture liaison service (FLS) programs facilitate bone health assessment following osteoporotic fractures and improve the rates of osteoporosis treatment initiation, compliance, and mortality [18, 19]. In 2014, our institution initiated a post-hip fracture FLS, and since July 2015, osteoporosis treatment has been offered to patients admitted to the geriatric ward for rehabilitation following a hip fracture.

## Purpose

We retrospectively investigate the incidence of specific AEs following in-hospital parenteral treatment with ZOL or Dmab in patients admitted for rehabilitation following a hip fracture, and we aim to identify risk factors for these AEs.

## Methods

### Study Population

This is a retrospective cohort study of patients insured by Clalit Health Services over the age of 65, who were hospitalized in Soroka University Medical Center in the geriatric ward after operative treatment of a low-trauma hip fracture between July 2015 and January 2020. Patients who were assessed by our institutional FLS and treated with ZOL or Dmab were included in this cohort. The choice of post-hip fracture antiosteoporosis treatment was made by the consulting endocrinologists based on current international guidelines. Generally, treatment-naïve patients who had eGFR > 35 mL/min were treated with ZOL, whereas patients who were treated with bisphosphonate prior to the hip fracture or had a eGFR between 20 and 35 mL/min were treated with Dmab. Patients on dialysis were excluded. All patients with serum 25-hydroxyvitamin D (25)OH(D) below 50 nmol/L were pretreated with vitamin D supplementation prior to the administration of ZOL or Dmab. Typically, if vitamin D levels were found to be below 50 nmol/L, 2 loading doses of vitamin D were prescribed at a dose of 40 000 IU each, and these were administered 1 week apart. If vitamin D levels were found to be between 50 and 75 nmol/L, 2000 IU of vitamin D was prescribed daily. Calcium was prescribed at a dose of 600 to 1200 mg daily. The dosage was determined following an assessment of enteral calcium intake and according to the pretreatment calcium level.

### Data Captured

Data was collected from hospital and community electronic medical records and included: demographic data (age and sex) and clinical data (body weight and body temperature). Clinical pretreatment interventions included the provision of paracetamol, dipyrone, hydration, calcium, and vitamin D supplementation. Laboratory data included serum 25)OH(D, measured using an immunoluminometric assay on a DiaSorin Liaison XL analyzer (RRID:AB_2811287), PTH measured using an immunoassay on a Siemens Advia Centaur system (RRID:AB_3271518), creatinine, albumin, and calcium. Pretreatment 25)OH(D and PTH serum concentrations were used when they were obtained up to 6 months prior to hospitalization for the index fracture. eGFR was calculated using the MDRD equation (186 × [serum creatinine (mg/dL)]^−1.154^ × (age)^−0.203^ × (0.742 if female).

### Primary Outcomes

Fever was defined as rise in body temperature >38 °C during the 48 hours following drug administration. Hypocalcemia was defined as albumin-corrected serum calcium less than 8.5 mg/dL during the 30 days following treatment. When albumin levels were not available (n = 97 of 362 patients included in study), they were assumed to be in the normal range. Renal function impairment was defined as an increase of 0.5 mg/dL or more in creatinine levels during the 30 days following treatment. This definition is in accordance with the definition of renal functional impairment used in previous studies [20].

### Secondary Outcomes

Secondary outcomes included demographic clinical and biochemical parameters that were associated with the primary outcome.

### Statistical Analysis

Continuous normally distributing variables are presented as means with SDs. Continuous variables that are not normally distributed are presented as medians with interquartile ranges. Categorical variables are presented as numbers and percentages. Differences in temperature, blood calcium, and creatinine levels before and after treatments were evaluated using Student's *t*-test or one-way ANOVA for continuous variables with normal distribution, Mann–Whitney U test (Wilcoxon rank sum test), or Kruskal–Wallis (one-way ANOVA on ranks) for continuous variables with nonnormal distribution and chi-square test for categorical data. Statistical significance was defined by a 2-sided *P*-value ≤ .05.

To evaluate the effect of the medications on body temperature, serum calcium levels, and creatinine levels, multivariable analyses using a linear mixed model were used. Estimate points with 95% confidence interval (CI) were used as a measure of association in the analysis. Statistically significant variables (*P*-value ≤.05) and clinically significant variables such as diabetes mellitus and other significant comorbidities were entered into the model. Goodness of fit was assessed by receiver operating characteristic analysis, and the final model was chosen through statistical significance and a reasonable clinical explanation. In addition to the linear mixed model, due to the nature of the database (repeated measurements), mixed effects logistic regression was also performed. As the outcome variable was dichotomized, odds ratios (ORs) with 95% CI were reported. All analyses were carried out using IBM SPSS Statistics software (Version 25).

## Results

### Study Population

Between July 2015 and January 2020, 614 patients over the age of 65 were hospitalized for rehabilitation in the Soroka University Medical Center geriatrics ward following surgery for a hip fracture. Two hundred fifty-three patients were excluded as they were not treated with ZOL or Dmab, for reasons that included acute illness, severe vitamin D deficiency, dialysis, and refusal to take the medication. Three hundred sixty-two patients met the inclusion criteria and, of those, 228 (63%) were treated with ZOL and 134 (37%) with Dmab.

Baseline characteristics of the study population are presented in Table 1. As expected, mean age was significantly higher in patients treated with Dmab (mean age 85 ± 7 vs 80 ± 7 *P* < .001), mean eGFR was lower (57 ± 25vs 80 ± 24 *P* < .001), and median PTH level was higher [84 (54-139) vs 65 (44-99) *P* < .001] compared with those treated with ZOL. Median baseline 25)OH(D and calcium levels did not differ between the 2 groups.

**Table 1.** Baseline characteristics of the study population according to postfracture osteoporosis treatment

	ZOL (n = 228)	Dmab (n = 134)	Missing (n)	*P*-value
Female: n (%)	160 (70)	89 (66)	0	.46
Age (years): mean ± SD	80 ± 7	85 ± 7	0	<.001
Calcium(mg\dl) mean ± SD	9.5 (9.2-9.7)	9.4 (9.2-9.7)	52	.51
eGFR (mL/min/1.73 m2): mean ± SD	80 ± 24	57 ± 25	3	<.001
25-hydroxyvitamin D (nmol/L): median (IQR)	47 (31-67)	49 (32-68)	17	.70
PTH level (pg\dl): median (IQR)	65 (44-99)	84 (54-139)	24	<.001

Abbreviations: Dmab, denosumab; eGFR, estimated glomerular filtration rate; IQR, interquartile range; ZOL, zoledronic acid.

Almost all patients were treated with vitamin D and calcium supplementation prior to the administration of ZOL or Dmab. A minority of Dmab patients received pretreatment hydration, and about 60% received pretreatment antipyrectics. Almost all ZOL patients received pretreatment hydration and antipyretic therapy. The median time from fracture to antiosteoporosis treatment did not differ between the 2 medication groups: 18 days for ZOL and 18.5 days for Dmab, *P* = .16.

### Fever

The rates of AEs are summarized in Table 2. The mean documented increase in body temperature 48 hours after ZOL administration was small (0.18 °C, *P* < .001) and there was no change in temperature after Dmab administration. Fever occurred in 20 (9%) patients following ZOL administration. In this group, 55% were female, and the mean age was 77.4 years. The mean maximal temperature was 38.7 °C, and the mean length of fever was 1.65 days. Most of these patients had concomitant flu-like symptoms. One patient had a concomitant surgical wound infection, and 4 patients had urinary tract infection. An alternative source of fever was not detected in the remaining 15 (75%) patients. Fever was not detected in the Dmab-treated group.

**Table 2.** Side effects of parenteral antiosteoporosis treatment following hip fracture

	ZOL (n = 228)	Dmab (n = 134)	*P*-value
Fever (temperature > 38°C) within 48 hours of treatment: n (%)	20 (9)	0 (0)	.001
Hypocalcemia (albumin corrected calcium <8.5 mg/dL) within 1 month of treatment: n (%)	41 (18)	39 (29)	.009
Severe hypocalcemia (albumin corrected calcium <7.5 mg/dL) within 1 month of treatment: n (%)	5 (2)	8 (6)	.06
Renal injury (serum creatinine elevation of >0.5 mg/dL) within 1 month of treatment: n (%)	9 (4)	6 (5)	0.81

Abbreviations: Dmab, denosumab; ZOL, zoledronic acid.

### Hypocalcemia

Hypocalcemia occurred in 41 (18%) and 39 (29%) patients treated with ZOL and Dmab, respectively (*P* = .009), which was detected on routine biochemical follow-up. Calcium levels decreased by a mean 0.6 and 0.9 mg/dL within 30 days after administration of ZOL and Dmab, respectively (*P* < .001). The clinical and biochemical characteristics of the study population according to posttreatment hypocalcemia status are presented in Table 3. Albumin levels were missing for 97 of 362 patients and were assumed to be normal. Patients who developed hypocalcemia had lower mean baseline calcium levels [median 9.3 mg/dL (9.1-9.6) vs 9.5 mg/dL (9.3-9.8) *P* < .001] and a lower mean eGFR (64 ± 29 mL/min vs 74 ± 25 mL/min *P* < .002). Mean pretreatment 25)OH(D levels did not differ between both groups. The mean length of hospitalization was longer in patients who developed hypocalcemia compared to those with normal mean calcium levels (24.9 ± 11.3 days vs 22.2 ± 7.3 days *P* = .02). Patients with hypocalcemia were more likely to have received Dmab than ZOL as compared with patients with normal mean calcium levels (49% vs 32% *P* < .009). The median length of time from fracture to treatment with ZOL or Dmab did not differ between patients who developed hypocalcemia compared to those who did not [19 days (15-25) vs 17 days (14-21) *P* = .07], respectively.

**Table 3.** **Characteristics of patients who developed hypocalcemia (corrected calcium < 8**.5 **mg/dL) within 30 days of treatment compared with patients with normal calcium levels**

	Hypocalcemia (n = 80)	Normal calcium levels (n = 220)	*P*-value
Female: n (%)	51 (64)	156 (71)	.24
Age, years: mean ± SD	83 ± 6	82 ± 8	.26
Pretreatment with calcium: n (%)	79 (9)	209 (95)	.17
Pretreatment with vitamin D: n (%)	79 (99)	216 (98)	.94
Baseline calcium level (mg/dL): median (IQR)	9.3 (9.1-9.6)	9.5 (9.3-9.8)	.001
Baseline 25-hydroxyvitamin D (nmol/L): median (IQR)	45 (31-66)	49 (33-68)	.28
Baseline eGFR (mL/min/1.73 m2): mean ± SD	64 ± 29	74 ± 25	.002
Hospitalization length, days: mean ± SD*	25 ± 11	22 ± 7	.02
Denosumab: n (%)	39 (49)	71 (32)	.009
Days between fracture and treatment: median (IQR)	19 (15-25)	17 (14-21)	0.7

Abbreviations: eGFR; estimated glomerular filtration rate, IQR; interquartile range.

Multivariable analysis of risk factors for posttreatment hypocalcemia, which adjusted for pretreatment serum calcium level, eGFR on admission, age, female sex, PTH level on admission, and the administration of Dmab, is presented in Table 4. Only pretreatment serum calcium independently predicted hypocalcemia (OR 0.30, 95% CI 0.13-0.68, *P* = .004), such that a decrease of 1 mg\dl from baseline calcium of 9.3 mg/dL increased the risk of hypocalcemia by 3.3-fold.

**Table 4.** Risk factors for hypocalcemia following drug administration using multivariable analysis and adjusted for pretreatment serum calcium level, eGFR on admission, age, sex, PTH level on admission, and the administration of denosumab

	OR	CI	*P*-value
Corrected calcium on admission	0.30	0.13-0.68	.004
eGFR on admission	0.99	0.98-1.01	.27
Age	1.00	0.96-1.05	.94
Sex (female)	0.84	0.44-1.61	.61
PTH level on admission	1.00	1.00-1.01	.12
Drug (denosumab)	1.38	0.70-2.74	.35

Abbreviations: CI, confidence interval; eGFR, estimated glomerular filtration rate; OR, odds ratio.

Severe hypocalcemia (<7.5 mg\dl) occurred in 5 patients (2%) after ZOL and 8 patients (6%) after Dmab treatment (*P* = .06) using calcium levels that had been corrected for serum albumin. Most of these patients had impaired renal function; 6 out of 13 patients had creatinine levels above 2 mg\dl. Six of the patients with severe hypocalcemia remained hospitalized within 1 month of antiosteoporosis treatment, but the cause was hypocalcemia in only 1 patient. These hospitalized patients had no documented symptoms of hypocalcemia or changes in electrocardiogram. Two patients continued to have hypocalcemia 1 year after treatment with Dmab.

### Renal Functional Impairment

ZOL administration was associated with a mean increase of serum creatinine by 0.1 mg/dL, *P* = .06. Dmab injection was not associated with a significant change in renal function. Renal functional impairment developed in 9 (4%) and 6 (5%) of patients treated with ZOL and Dmab, respectively (*P* = .81). Ten out of 15 (67%) patients whose serum creatinine increased by at least 0.5 mg/dL were male (*P* = .004). No other significant differences were detected between the 2 groups including age, baseline eGFR, pretreatment hydration, and time from fracture to treatment. Eight patients had an increase of creatinine above 2 mg/dL, which occurred 1 to 7 days following treatment, 4 patients in each drug treatment group. Creatinine levels returned to baseline in 5 of these patients within 1 month. One patient developed CKD after treatment with ZOL and also suffered from posttreatment fever and hypocalcemia. Two patients died during hospitalization due to other complications. Both univariate analysis and multivariable analysis adjusted for eGFR on admission, Dmab administration, age, female sex, and pretreatment hydration showed that female sex was associated with a lower risk of renal functional impairment following either medication during 30 days of follow-up (OR 0.25, 95% CI 0.08-0.81, *P* = .02) (Supplementary Tables 1 and 2) [21].

## Discussion

In this retrospective study, we evaluated the safety of FLS-based, in-hospital treatment with ZOL and Dmab following surgical repair of a hip fracture in patients aged over 65 years. Fever was uncommon after ZOL, and hypocalcemia was relatively common after administration of either ZOL or Dmab. Renal function deterioration was rare after either ZOL or Dmab.

Fever is a well-established side effect of ZOL treatment but not of Dmab. It may be accompanied by flu-like symptoms (arthralgia, myalgia, and headache) as part of an APR [22]. Analysis of the APR that developed among HORIZON study participants found that fever occurred in 17% and 2% in the ZOL and placebo arms, respectively [23]. A recent study that measured patients’ temperature on consecutive days after ZOL showed that administration of acetaminophen for at least 48 hours following ZOL infusion significantly reduced the rate of posttreatment fever (4% vs 15%, respectively, *P* < .05) and the average increase in temperature (0.2 vs 0.4 °C, respectively, *P* < .05) [24]. Our results were similar [20 patients (9%) experienced fever, and the average temperature rise was 0.18 °C], which we attribute to using a pretreatment protocol that included antipyretic therapy for 48 hours following ZOL administration, which was not part of the protocol in the HORIZON study.

In the HORIZON recurrent fracture trial [4] and FREEDOM extension [5] studies, the rate of hypocalcemia was less than 1% in patients who received ZOL and Dmab, respectively, with no reported cases of symptomatic hypocalcemia. However, real-life studies have shown a higher prevalence of 6.3% to 7.4%, which correlated with a reduction in eGFR and poor nutritional status [25, 26]. The rate of hypocalcemia in our study was relatively high (18% after ZOL and 29% after Dmab). Possible contributing factors include longer follow-up (patients were followed up for 1 month in our study vs 11 and 10 days in the HORIZON and FREEDOM extension study, respectively [5, 27]), a higher threshold for reporting hypocalcemia (8.5 mg/dL, equivalent to 2.12 mmol/L was used in our study vs less than 2.1 mmol/L in the HORIZON study [27]), and other factors such as concomitant infection or the administration of other medications (like diuretics) or blood transfusions.

Severe hypocalcemia has barely been reported in controlled trials [4, 5, 24] and is therefore probably very rare. In our study, severe hypocalcemia [albumin corrected calcium of <7.5 mg\dl) occurred in 5 patients (2%) after ZOL and in 8 patients (6%) after Dmab treatment (*P* = .06)]. Longer follow-up may have been a contributing factor for the relatively high rates of severe hypocalcemia seen in our cohort. It is also possible that some of the patients who were found to be deficient in vitamin D were inadequately treated, as vitamin D levels were not rechecked to ensure adequate replacement following the loading doses. Persistent vitamin D deficiency may have contributed to the development of severe hypocalcemia.

Two patients continued to have hypocalcemia 1 year after treatment with Dmab, possibly due to the administration of additional doses, which are usually given biannually.

On univariate analysis, baseline calcium levels, baseline eGFR, length of hospitalization, and treatment with Dmab were associated with posttreatment hypocalcemia. On multivariate analysis, only baseline calcium levels were associated with hypocalcemia, such that a decrease of 1 mg/dL from baseline calcium of 9.3 mg/dL was associated with an increased risk of hypocalcemia by 3.3-fold. We hypothesize that lower baseline calcium levels reflect the net effect of several risk factors that become significant only when combined, including baseline renal function, vitamin D status, calcium intake, and gastrointestinal absorption capability. Interestingly, baseline calcium levels in the group with posttreatment hypocalcemia were in the middle section of the normal reference range. Several other studies in Dmab-treated patients found baseline calcium levels in the same range as our study (9.3 and 9.1 mg/dL) to be a predictor for Dmab-induced hypocalcemia [25, 28].

On univariate analysis, it was found that Dmab uptake was higher in patients who subsequently experienced treatment-induced hypocalcemia compared to patients with normocalcemia [39 (49%) vs 71 (32%) *P* = .009]. While the absolute risk of hypocalcemia was higher in the Dmab group, multivariate analysis did not find that the choice of drug was predictive of hypocalcemia. This may reflect patient selection such that patients who were older and had a lower eGFR and therefore at greater risk of developing treatment-induced hypocalcemia were more likely to be administered Dmab in lieu of ZOL.

There is a paucity of data in the literature regarding the association between the timing of antiresorptive treatment administration in relation to hip fracture and hypocalcemia risk [29]. We found that patients who developed posttreatment hypocalcemia received antiosteoporosis treatment on average 2 days later than those who did not develop hypocalcemia (19 vs 17 days, *P* = .07). The same was demonstrated for patients who developed severe hypocalcemia. On multivariate analysis, the timing of antiosteoporosis treatment was not associated with posttreatment hypocalcemia.

In the HORIZON study, a significant transient increase in serum creatinine was observed (1.3% vs 0.4% in ZOL and placebo group, respectively) [27]. This effect had no clinical significance. Similar results were noted in the HORIZON extension study and other studies [24, 30-32]. Studies with patients who received Dmab did not show any significant change in renal function [33, 34]. In our study, low rates of renal functional impairment were observed. eGFR deteriorated significantly in patients who received ZOL but not Dmab, although this was clinically insignificant. Because of the low incidence and the fact that renal functional impairment occurred during 30 days of follow-up following antiosteoporotic treatment, it is difficult to establish causality as other factors may have impacted renal function during this long follow-up period.

Our study has several limitations. First, the retrospective nature of the study led to missing data. Albumin levels were missing for 97 of 362 patients. We chose to assume that albumin levels were normal, but this may have led to overestimation of the rates of hypocalcemia following treatment with both antiosteoporosis medications. In addition, the frequency of blood testing was inconsistent. We addressed this issue by comparing minimum, maximum, and mean blood test results. There was a lack of data regarding patients’ comorbidities, which may have influenced the AEs of interest, and only partial data regarding clinical symptoms and clinical sequelae of patients with severe hypocalcemia and severe renal injury were available. Finally, the antiosteoporosis medications were given on average 18 and 18.5 days (ZOL and Dmab, respectively) following hip fracture and were given in an inpatient setting. The average length of hospital stay following hip fracture may be shorter in other countries, which might prevent the administration of these drugs in an inpatient setting and therefore limits the generality of our data [35].

This study benefitted from being a real-life study that was conducted in a large, single tertiary center with access to in-hospital and outpatient lab results.

## Conclusion

Parenteral osteoporosis treatment with either ZOL or Dmab in elderly patients post-hip fracture in a hospital setting is relatively safe if administered according to established international guidelines and with appropriate premedication including antipyretic therapy and hydration. Hypocalcemia is the most significant complication, and we suggest careful assessment of calcium and vitamin D status prior to treatment and that treatment postponement be considered in patients with baseline calcium levels below 9.3 mg/dL. The risk of posttreatment hypocalcemia does not differ between patients who receive ZOL or Dmab after adjustment for multiple confounders.

## Data Availability

Restrictions apply to the availability of some, or all data generated or analyzed during this study to preserve patient confidentiality or because they were used under license. The corresponding author will on request detail the restrictions and any conditions under which access to some data may be provided.

## References

[1] Pekonen S-R, Kopra J, Kröger H, Rikkonen T, Sund R. Regional and gender-specific analyses give new perspectives for secular trend in hip fracture incidence. Osteoporos Int. 2021;32(9):1725‐1733. 33712877 10.1007/s00198-021-05906-6 PMC8387269

[2] Lorentzon M . Treating osteoporosis to prevent fractures: current concepts and future developments. J Intern Med. 2019;285(4):381‐394. 30657216 10.1111/joim.12873

[3] Management of osteoporosis in postmenopausal women: the 2021 position statement of the North American Menopause Society. Menopause. 2021;28(9):973‐997. 34448749 10.1097/GME.0000000000001831

[4] Lyles KW, Colón-Emeric CS, Magaziner JS, et al Zoledronic acid and clinical fractures and mortality after hip fracture. N Engl J Med. 2007;357(18):1799‐1809. 17878149 10.1056/NEJMoa074941 PMC2324066

[5] Bone HG, Wagman RB, Brandi ML, et al 10 years of denosumab treatment in postmenopausal women with osteoporosis: results from the phase 3 randomised FREEDOM trial and open-label extension. Lancet Diabetes Endocrinol. 2017;5(7):513‐523. 28546097 10.1016/S2213-8587(17)30138-9

[6] Sieber P, Lardelli P, Kraenzlin CA, Kraenzlin ME, Meier C. Intravenous bisphosphonates for postmenopausal osteoporosis: safety profiles of zoledronic acid and ibandronate in clinical practice. Clin Drug Investig. 2013;33(2):117‐122. 10.1007/s40261-012-0041-1 23184667

[7] Adami S, Bhalla AK, Dorizzi R, et al The acute-phase response after bisphosphonate administration. Calcif Tissue Int. 1987;41(6):326‐331. 3124942 10.1007/BF02556671

[8] Huynh ALH, Baker ST, Stewardson AJ, Johnson DF. Denosumab-associated hypocalcaemia: incidence, severity and patient characteristics in a tertiary hospital setting. Pharmacoepidemiol Drug Saf. 2016;25(11):1274‐1278. 27255807 10.1002/pds.4045

[9] Black DM, Reid IR, Cauley JA, et al The effect of 6 versus 9 years of zoledronic acid treatment in osteoporosis: a randomized second extension to the HORIZON-Pivotal Fracture Trial (PFT). J Bone Miner Res. 2015;30(5):934‐944. 25545380 10.1002/jbmr.2442

[10] Wark JD, Bensen W, Recknor C, et al Treatment with Acetaminophen/paracetamol or ibuprofen alleviates post-dose symptoms related to intravenous infusion with zoledronic acid 5 mg. Osteoporos Int. 2012;23(2):503‐512. 21331467 10.1007/s00198-011-1563-8

[11] Al Elq AH . Symptomatic hypocalcemia associated with zoledronic acid treatment for osteoporosis: a case report. Oman Med J. 2013;28(2):e043. 31435469 10.5001/omj.2013.42 PMC6667807

[12] Morony S, Warmington K, Adamu S, et al The inhibition of RANKL causes greater suppression of bone resorption and hypercalcemia compared with bisphosphonates in two models of humoral hypercalcemia of malignancy. Endocrinology. 2005;146(8):3235‐3243. 15845617 10.1210/en.2004-1583

[13] Ominsky MS, Libanati C, Niu Q-T, et al Sustained modeling-based bone formation during adulthood in cynomolgus monkeys may contribute to continuous BMD gains with denosumab. J Bone Miner Res. 2015;30(7):1280‐1289. 25684625 10.1002/jbmr.2480

[14] Ho JW, Sundar S. Prolonged hypocalcemia after zoledronic acid in a patient with metastatic prostate carcinoma: did zoledronic acid trigger osteoblastic activity and avid calcium uptake? Clin Genitourin Cancer. 2012;10(1):50‐53. 22245101 10.1016/j.clgc.2011.11.004

[15] Hirschberg R . Renal complications from bisphosphonate treatment. Curr Opin Support Palliat Care. 2012;6(3):342‐347. 22710581 10.1097/SPC.0b013e328356062e

[16] Papapetrou PD . Bisphosphonate-Associated adverse events. Hormones (Athens). 2009;8(2):96‐110. 19570737 10.14310/horm.2002.1226

[17] Miller PD . The kidney and bisphosphonates. Bone. 2011;49(1):77‐81. 21232648 10.1016/j.bone.2010.12.024

[18] Walters S, Khan T, Ong T, Sahota O. Fracture liaison services: improving outcomes for patients with osteoporosis. Clin Interv Aging. 2017;12:117‐127. 28138228 10.2147/CIA.S85551 PMC5237590

[19] Fan W, Machado M, Leder BZ, et al Inpatient zoledronic acid and integrated orthopedic and fracture liaison services improve osteoporosis treatment rates. J Clin Endocrinol Metab. 2022;108(1):191‐197. 36056816 10.1210/clinem/dgac508

[20] Rosen LS, Gordon D, Kaminski M, et al Zoledronic acid versus pamidronate in the treatment of skeletal metastases in patients with breast cancer or osteolytic lesions of multiple myeloma: a phase III, double-blind, comparative trial. Cancer J. 2001;7:377‐387. 11693896

[21] Abu-Jwead A, Fisher DL, Goldabart A, et al Figshare Digital Repository. 2024. doi:10.6084/m9.figshare.26319025 Date of deposit 17 July 2024.

[22] Kotian P, Boloor A, Sreenivasan S. Study of adverse effect profile of parenteral zoledronic acid in female patients with osteoporosis. J Clin Diagn Res. 2016;10(1):OC04‐OC06. 10.7860/JCDR/2016/17061.7021 PMC4740633 26894105

[23] Reid IR, Gamble GD, Mesenbrink P, Lakatos P, Black DM. Characterization of and risk factors for the acute-phase response after zoledronic acid. J Clin Endocrinol Metab. 2010;95(9):4380‐4387. 20554708 10.1210/jc.2010-0597

[24] Fan W, Leder BZ, Mannstadt M, Ly TV, Franco-Garcia E, Bolster MB. Safety of inpatient zoledronic acid in the immediate postfracture setting. J Clin Endocrinol Metab. 2023;108(11):e1282‐e1288. 37227016 10.1210/clinem/dgad295

[25] Tsvetov G, Amitai O, Shochat T, Shimon I, Akirov A, Diker-Cohen T. Denosumab-Induced hypocalcemia in patients with osteoporosis: can you know who will get low? Osteoporos Int. 2020;31(4):655‐665. 31838550 10.1007/s00198-019-05261-7

[26] Spångeus A, Rydetun J, Woisetschläger M. Prevalence of denosumab-induced hypocalcemia: a retrospective observational study of patients routinely monitored with ionized calcium post-injection. Osteoporos Int. 2024;35(1):173‐180. 37750930 10.1007/s00198-023-06926-0 PMC10786736

[27] Black DM, Delmas PD, Eastell R, et al Once-Yearly zoledronic acid for treatment of postmenopausal osteoporosis. N Engl J Med. 2007;356(18):1809‐1822. 17476007 10.1056/NEJMoa067312

[28] Chen J, Smerdely P. Hypocalcaemia after denosumab in older people following fracture. Osteoporos Int. 2017;28(2):517‐522. 27682248 10.1007/s00198-016-3755-8

[29] Conley RB, Adib G, Adler RA, et al Secondary fracture prevention: consensus clinical recommendations from a multistakeholder coalition. J Bone Miner Res. 2020;35(1):36‐52. 31538675 10.1002/jbmr.3877

[30] Black DM, Reid IR, Boonen S, et al The effect of 3 versus 6 years of zoledronic acid treatment of osteoporosis: a randomized extension to the HORIZON-pivotal fracture trial (PFT). J Bone Miner Res. 2012;27(2):243‐254. 22161728 10.1002/jbmr.1494 PMC3427916

[31] Laxminarayan R, Sayed N, Nisar M, Swamy G, Rachael H. THU0484 zoledronic acid infusion and effect on renal function and calcium in osteoporosis. Ann Rheum Dis. 2016;75(Suppl 2):366‐367.

[32] Fixen CW, Fixen DR. Renal safety of zoledronic acid for osteoporosis in adults 75 years and older. Osteoporos Int. 2022;33(11):2417‐2422. 35829757 10.1007/s00198-022-06499-4

[33] Ohishi T, Fujita T, Nishida T, Hagiwara K, Murai R, Matsuyama Y. Effect of denosumab on renal function in women with osteoporosis evaluated using cystatin C. Osteoporos Sarcopenia. 2022;8(2):68‐74. 35832419 10.1016/j.afos.2022.05.002 PMC9263171

[34] Broadwell A, Chines A, Ebeling PR, et al Denosumab safety and efficacy among participants in the FREEDOM extension study with mild to moderate chronic kidney disease. J Clin Endocrinol Metab. 2021;106(2):397‐409. 33211870 10.1210/clinem/dgaa851 PMC7823314

[35] Nikkel LE, Kates SL, Schreck M, Maceroli M, Mahmood B, Elfar JC. Length of hospital stay after hip fracture and risk of early mortality after discharge in New York state: retrospective cohort study. BMJ. 2015;351:h6246. 26655876 10.1136/bmj.h6246 PMC4674667

